# Statistical Analysis of Telehealth Use and Pre- and Postpandemic Insurance Coverage in Selected Health Care Specialties in a Large Health Care System in Arkansas: Comparative Cross-Sectional Study

**DOI:** 10.2196/49190

**Published:** 2024-10-18

**Authors:** Aysenur Betul Cengil, Sandra Eksioglu, Burak Eksioglu, Hari Eswaran, Corey J Hayes, Cari A Bogulski

**Affiliations:** 1 Industrial Engineering Department College of Engineering University of Arkansas Fayetteville, AR United States; 2 Department of Obstetrics and Gynecology College of Medicine University of Arkansas for Medical Sciences Little Rock, AR United States

**Keywords:** appointment scheduling metrics, insurance coverage, statistical hypothesis testing, telehealth

## Abstract

**Background:**

The COVID-19 pandemic triggered policy changes in 2020 that allowed insurance companies to reimburse telehealth services, leading to increased telehealth use, especially in rural and underserved areas. However, with many emergency rules ending in 2022, patients and health care providers face potential challenges in accessing these services.

**Objective:**

This study analyzed telehealth use across specialties in Arkansas before and after the pandemic (2017-2022) using data from electronic medical records from the University of Arkansas for Medical Sciences Medical Center. We explored trends in insurance coverage for telehealth visits and developed metrics to compare the performance of telehealth versus in-person visits across various specialties. The results inform insurance coverage decisions for telehealth services.

**Methods:**

We used pre- and postpandemic data to determine the impacts of the COVID-19 pandemic and changes in reimbursement policies on telehealth visits. We proposed a framework to calculate 3 appointment metrics: indirect waiting time, direct waiting time, and appointment length. Statistical analysis tools were used to compare the performance of telehealth and in-person visits across the following specialties: *obstetrics and gynecology*, *psychiatry*, *family medicine*, *gerontology*, *internal medicine*, *neurology*, and *neurosurgery*. We used data from approximately 4 million in-person visits and 300,000 telehealth visits collected from 2017 to 2022.

**Results:**

Our analysis revealed a statistically significant increase in telehealth visits across all specialties (*P*<.001), showing an 89% increase from 51,589 visits in 2019 to 97,461 visits in 2020, followed by a 21% increase to 117,730 visits in 2021. Around 92.57% (134,221/145,001) of telehealth patients from 2020 to 2022 were covered by *Medicare*, *Blue Cross* and *Blue Shield*, *commercial and managed care*, *Medicaid*, and *Medicare Managed Care*. In-person visits covered by *Medicare* and *Medicaid* decreased by 15%, from 313,196 in 2019 to 264,696 in 2022. During 2020 to 2022, about 22.84% (33,123/145,001) of total telehealth visits during this period were covered by *Medicare* and 53.58% (86,317/161,092) were in *psychiatry*, *obstetrics and gynecology*, and *family medicine*. We noticed a statistically significant decrease (*P*<.001) in the average indirect waiting time for telehealth visits, from 48.4 to 27.7 days, and a statistically significant reduction in appointment length, from 93.2 minutes in 2020 to 39.59 minutes in 2022. The indirect waiting time for *psychiatry* telehealth visits was almost 50% shorter than that for in-person visits. These findings highlight the potential benefits of telehealth in providing access to health care, particularly for patients needing psychiatric care.

**Conclusions:**

Reverting to prepandemic regulations could negatively affect Arkansas, where many live in underserved areas. Our analysis shows that telehealth use remained stable beyond 2020, with *psychiatry* visits continuing to grow. These findings may guide insurance and policy decisions in Arkansas and other regions facing similar access challenges.

## Introduction

### Background

Since 2020, there has been a significant increase in the use of telehealth services in the United States due to the COVID-19 pandemic, which was declared a public health emergency [1-4]. Telehealth visits have become an essential means of providing patients with access to care while reducing the risk of exposure for both patients and health care providers [5,6]. In response, the Health and Human Services Office for Civil Rights relaxed certain regulations, making it easier to provide health services via remote communication technology [7,8]. The Affordable Care Act also allowed Medicare and Medicaid to cover and reimburse the use of certain telehealth services, which further increased their use. In addition, the rapid growth of telecommunication technologies in recent years has facilitated the delivery of care via telehealth [9].

However, with the end of many emergency rules in 2022, federal government offices and private insurance companies are changing their reimbursement policies [10-12]. Thus, patients and health care providers are concerned about losing access to telehealth [13,14]. The rollback of COVID-19 waivers, coverage and payment policies, lack of insurer coverage of telehealth visits, and low or no reimbursement are among the top barriers to using telehealth, according to a recent survey of 1545 physicians by the American Medical Association [15]. Nevertheless, studies have shown that patients and health care providers are willing to use telehealth visits after the COVID-19 pandemic [16-19]. To evaluate the trends in telehealth use in Arkansas from 2017 to 2022, we conducted a study using data from the electronic medical records of the University of Arkansas for Medical Sciences Medical Center (UAMS Health), a network of health care providers across the state. Our analysis revealed that there continues to be a strong demand for telehealth services for certain specialties, such as psychiatry, obstetrics and gynecology (OB/GYN), and family medicine.

### Objectives

Our study aims to analyze trends in telehealth use across specialties and insurance coverage before and after the pandemic. The study also uses performance metrics, such as indirect waiting time, direct waiting time, and appointment length, to compare telehealth and in-person visits. We aim to identify the most popular specialties among telehealth users and highlight observations about the performance of telehealth visits within these specialties. By doing so, we aim to provide data-driven evidence of telehealth use in Arkansas that can be used by insurance companies and policy makers to reevaluate their telehealth reimbursement policies.

## Methods

### Study Design

We used the patient data collected from the Arkansas Clinical Data Repository, which is maintained by the Department of Biomedical Informatics in the College of Medicine at the University of Arkansas for Medical Sciences (UAMS). We conducted a time series comparative cross-sectional study to investigate insurance coverage trends before and after the pandemic for different specialties. We compared the performance of telehealth and in-person visits and evaluated telehealth performance over time for these specialties.

### Setting

**Figure 1:**
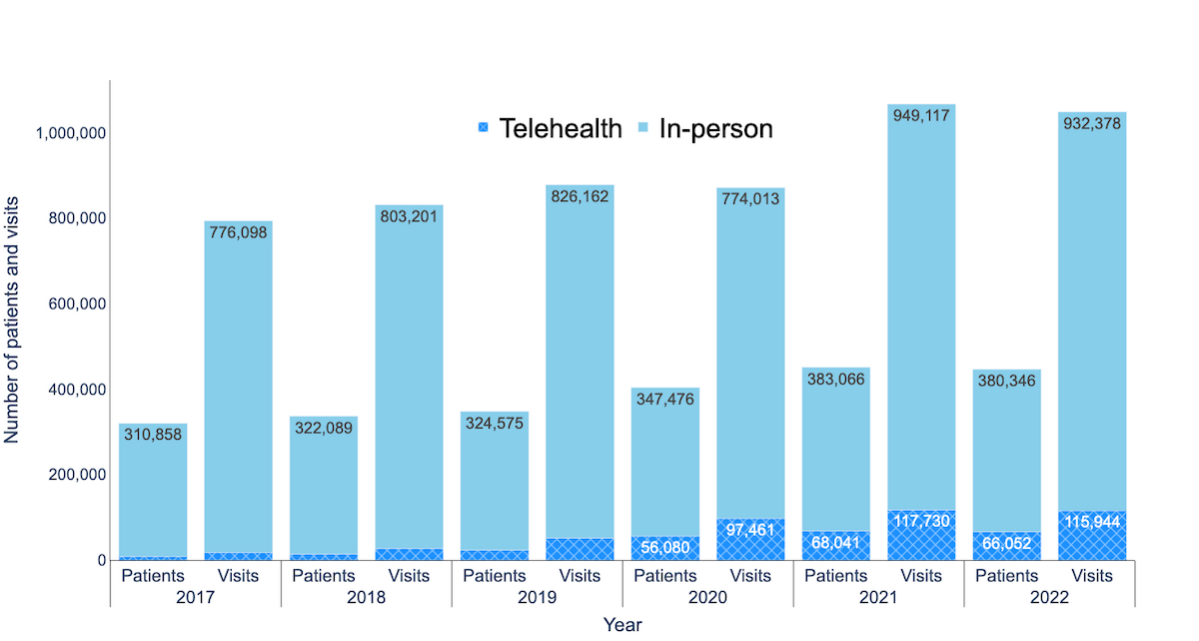
Number of patients and visits (2017-2022).

The Arkansas Clinical Data Repository collects demographic, inpatient and outpatient health care use, and pharmacy prescription–related data from electronic medical records. Figure 1 illustrates the number of patients and visits for all health care specialties from 2017 to 2022, including both telehealth and in-person appointments. The data analyzed were collected from patients residing in Arkansas and neighboring states who scheduled appointments at UAMS Health. The data include insurance coverage information for both in-person and telehealth visits and appointment time stamps, including appointment creation day, appointment day, check-in and checkout times, and roomed and visit end times.

### Data Preprocessing

#### Overview

We defined participants by indicating the inclusion and exclusion criteria for insurance coverage and appointment scheduling metrics. We have provided a detailed explanation of how the study size was determined. In addition, we have highlighted the outcomes and exposures for the analyses (insurance coverage and appointment scheduling metrics).

#### Insurance Coverage

In our analyses, we excluded visits that do not have insurance provider information. Table 1 summarizes the number and the corresponding percentage of visits without insurance provider information for in-person and telehealth visits. For in-person visits without insurance information, *radiology* accounts for 55.56% (347,386/625,295) visits of the observations, *family medicine* accounts for 12.91% (80,722/625,295), *radiation oncology* accounts for 11.94% (74,643/625,295), and 15.43% (96,477/625,295) do not have any specialty information. Among telehealth visits without insurance information, *call center* [20] accounts for 67.68% (184,989/273,309) of the observations, *OB/GYN* accounts for 19.5% (53,292/273,309), and *HealthNow* [21,22] accounts for 9.64% (26,339/273,309). It should be noted that patients are not billed for *call center* services.

**Table 1:** Number and percentage of in-person and telehealth visits without insurance provider information.

Year	In-person visits (n=625,295), n (%)	Telehealth visits (n=273,309), n (%)
2017	65,985 (8.5)	13,754 (79.45)
2018	83,074 (10.34)	24,746 (89.03)
2019	96,490 (11.68)	48,675 (94.35)
2020	79,594 (10.28)	57,944 (59.45)
2021	108,964 (11.48)	62,198 (52.83)
2022	191,188 (20.51)	65,992 (56.92)

**Figure 2:**
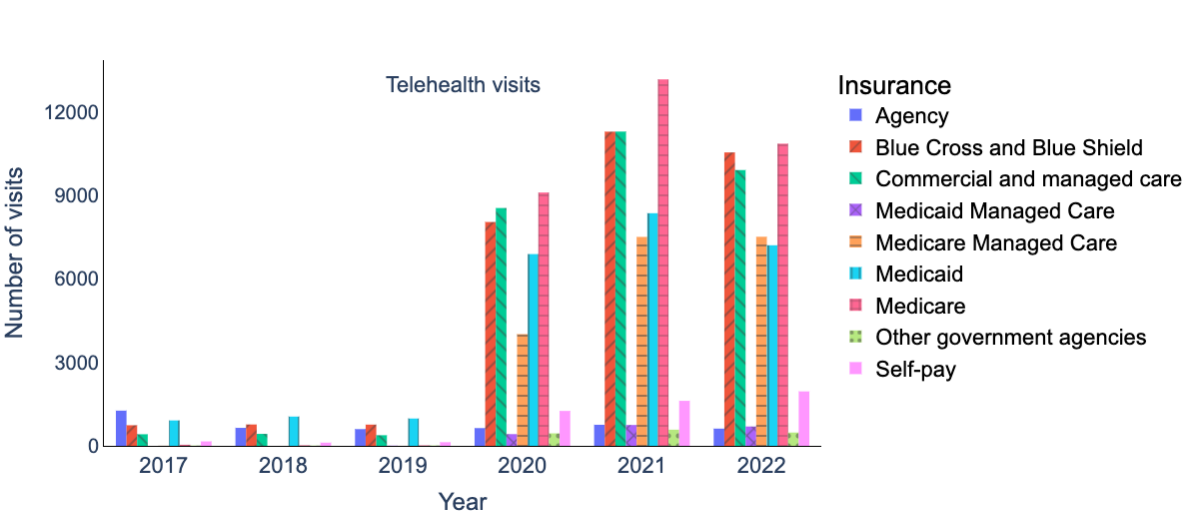
Insurance coverage breakdown (2017-2022) for telehealth visits.

Figures 2 and 3 present a breakdown of insurance coverage for telehealth and in-person visits from 2017 to 2022. Our analysis excluded the following insurance providers: *international*, *prepay non-Arkansas resident*, and *worker’s compensation*, as these insurance providers cover only 0.36% (564/154,523) and 1.06% (46,894/4,435,674) of total telehealth and in-person visits, respectively. After preprocessing the data, we evaluated the insurance coverage trends (our primary outcome) of telehealth visits over time. We identified top insurance providers for certain specialties (our secondary outcome) based on the number of telehealth visits over time (our exposures).

#### Appointment Scheduling

**Figure 4:**
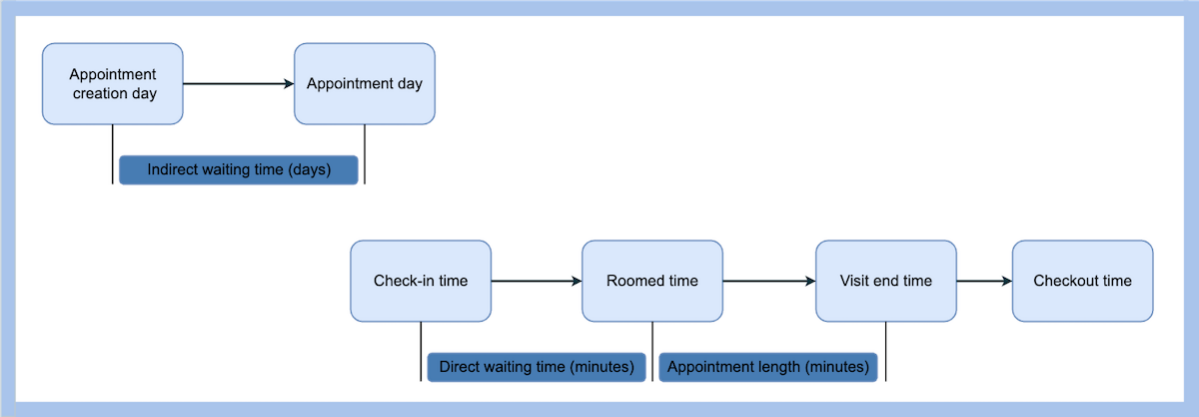
Workflow for both telehealth and in-person appointments.

The workflow diagram of appointment scheduling at UAMS Health, whether for telehealth or in-person visits, is presented in Figure 4. To evaluate the scheduling efficiency, we used 3 metrics: *indirect waiting time*, *direct waiting time*, and *appointment length*. Indirect waiting time refers to the number of days between the *appointment creation* and the *appointment day*. Direct waiting time represents the difference between *check-in* and *roomed time*, while appointment length is the duration between *roomed time* and *visit end time*, both measured in minutes. It is important to note that some visits may not have all the necessary time stamps to calculate these metrics.

**Figure 5:**
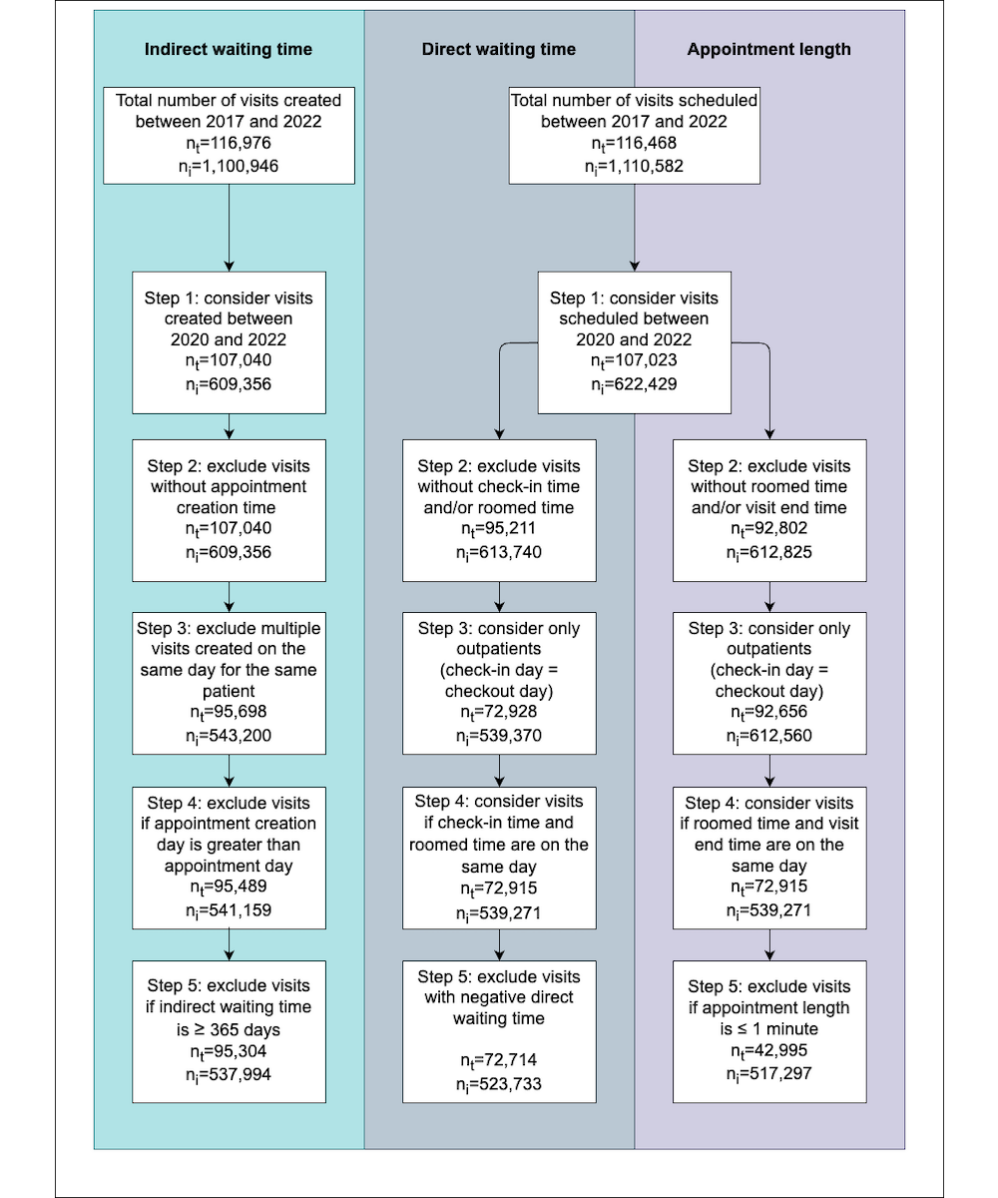
Flow diagrams of data extraction and analysis for each scheduling metric. n_i_: number of in-person visits; n_t_: number of teleheath visits.

The process used to extract the data, build the dataset, and calculate the scheduling metrics for our dataset is shown in Figure 5. We established rules to identify errors and outliers and removed visits with incomplete time stamps, errors, and outliers. The first column of Figure 5 provides an overview of the process for calculating the indirect waiting time metric. For this metric, we excluded visits without an appointment creation time and visits where the creation day is after the appointment day. In cases where a patient created multiple appointments on the same day, we only considered the earliest one. We also identified appointments with an indirect waiting time >365 days as outliers. Similar procedures were applied to calculate the direct waiting time and appointment length metrics. The total number of telehealth and in-person visits considered in each step of this process are presented in Figure 5. Using the subset of visits for each scheduling metric, we compared the performance of telehealth and in-person visits (our primary outcome) via different scheduling metrics (our exposures).

### Data Analysis: Statistical Analysis for Insurance Coverage

To determine whether there is a statistically significant change in insurance coverage, we conducted additional hypothesis tests. An example hypothesis we tested is the following:

- H_0_: There is no difference between the mean number of insurance-covered telehealth visits in 2019 and 2020.
- H_a_: There is a difference between the mean number of insurance-covered telehealth visits in 2019 and 2020.

We conducted similar hypothesis testing to compare insurance coverage for telehealth visits during 2020 and 2021 as well as 2021 and 2022. The same set of hypothesis tests was conducted for in-person visits.

We conducted tests to determine if the data are normally distributed. We used the paired 2-tailed *t* test for normally distributed data and the Wilcoxon signed rank test for nonnormally distributed data. The significance threshold (α) is .05 [23,24]. First, we used a 2-tailed test to evaluate if the population means are significantly different. For the case where the null hypothesis was rejected, we conducted additional 1-tailed tests to determine whether the difference in means is >0.

### Statistical Analysis for Appointment Scheduling Metrics

The objective of this analysis was to investigate whether there is a statistically significant difference in performance metrics between in-person and telehealth services from 2020 to 2022 and between consecutive years for telehealth (or in-person) services. As telehealth use differs among specialties, we focused on the specialties with the most telehealth visits during 2020 to 2022. We conducted several hypothesis testing using the processed data. Here is an example of one of the hypotheses tested for *OB/GYN* visits:

- H_0_: There is no difference between the means of direct waiting time for in-person and telehealth OB/GYN visits in 2020.
- H_a_: There is a difference between the means of direct waiting time for in-person and telehealth OB/GYN visits in 2020.

We performed similar hypothesis testing for indirect waiting time, direct waiting time, and appointment length for the years 2021 and 2022. In addition, we compared the appointment scheduling metrics for telehealth (in-person) visits in consecutive years and extended the hypothesis testing to other specialties. Because our sample size is large, we used a 2-tailed *z* test with a significance threshold (α) of .05 [23,24].

For those cases where the null hypothesis was rejected, we conducted additional tests. We performed 1-tailed *z* tests to determine whether the difference between the means is >0.

### Ethical Considerations

Data collection was approved by the Institutional Review Board of the UAMS (IRB 275271). The study was determined to not be human participant research. The data were deidentified, and there was no interaction with individuals.

## Results

### Trends in Telehealth and In-Person Visits

#### Telehealth Visits

**Figure 6:**
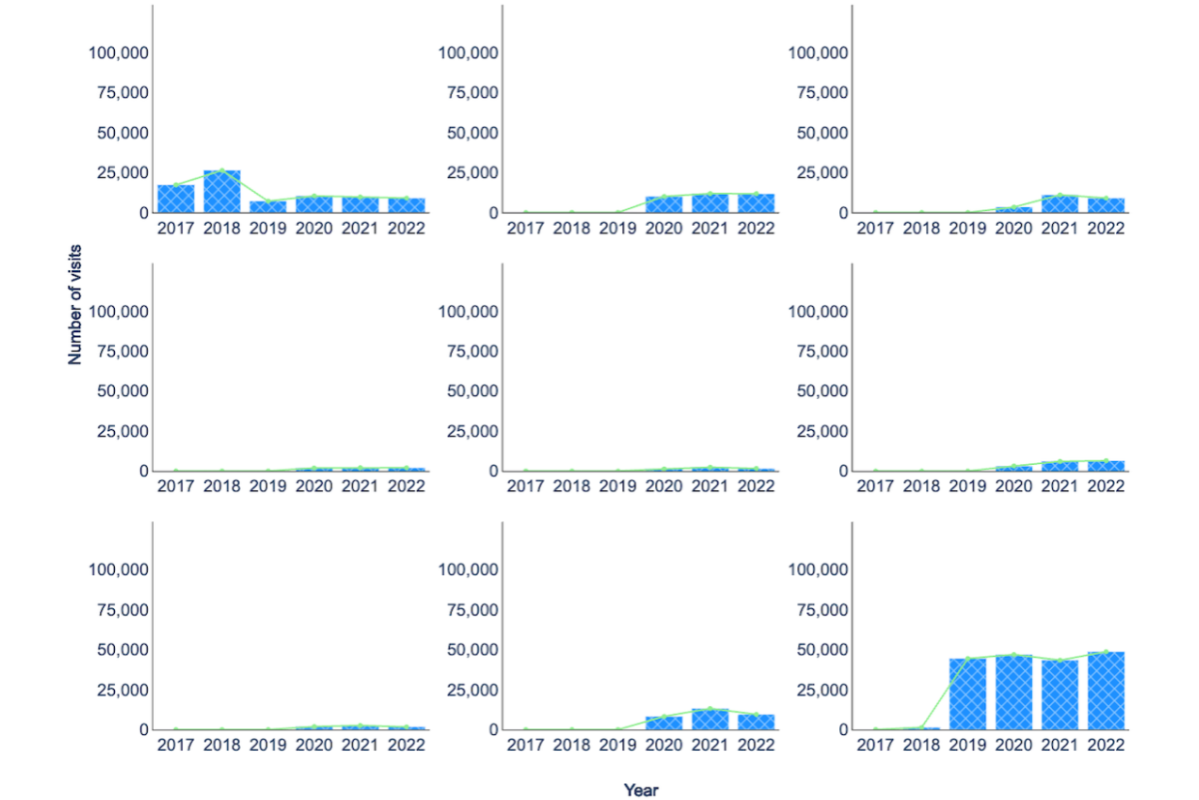
Number of visits for the selected health care specialties, call center and HealthNow (2017-2022) for telehealth visits. OB/GYN: obstetrician and gynecologist.

In Figure 6, we present data on the number of telehealth visits at UAMS Health from 2017 to 2022, covering 7 different specialties (*psychiatry*, OB*/*GYN, *family medicine*, *gerontology*, *internal medicine*, *neurology*, and *neurosurgery*) as well as the *call center* and *HealthNow*. Before 2020, telehealth services were only used by *OB/GYN* patients due to the Antenatal and Neonatal Guidelines, Education and Learning System (ANGELS) program, which began in 2003 to provide telemedicine consultations to pregnant women considered high risk at the UAMS [25]. The number of *OB/GYN* visits increased from 17,250 in 2017 to 26,381 in 2018. This corresponds to a 53% increase. However, this number decreased significantly in 2019. Between 2020 and 2022, these 7 specialties, along with the *call center* and *HealthNow*, accounted for almost 87.52% (289,829/331,135) of all telehealth visits. Interestingly, the corresponding number of in-person visits for these 7 specialties only represented about 21.95% (582,865/2,655,508) of the total in-person visits.

In 2020, telehealth visits experienced a substantial increase in response to the COVID-19 pandemic. As shown in Figure 6, in 2021, the number of telehealth visits for *family medicine* went from 3381 to 11,035, which corresponds to a 226.4% increase. The number of *neurology* visits went from 3175 to 6062, a 90.9% increase, and the number of *internal medicine* visits went from 1299 to 2407, an 85.3% increase. However, in 2022, telehealth visits decreased for all specialties, except *neurology*, with an accompanying rise in the use of the *call center*. This trend is consistent with the national decrease in telehealth use, possibly due to the decreasing number and severity of COVID-19 cases [26].

**Figure 7:**
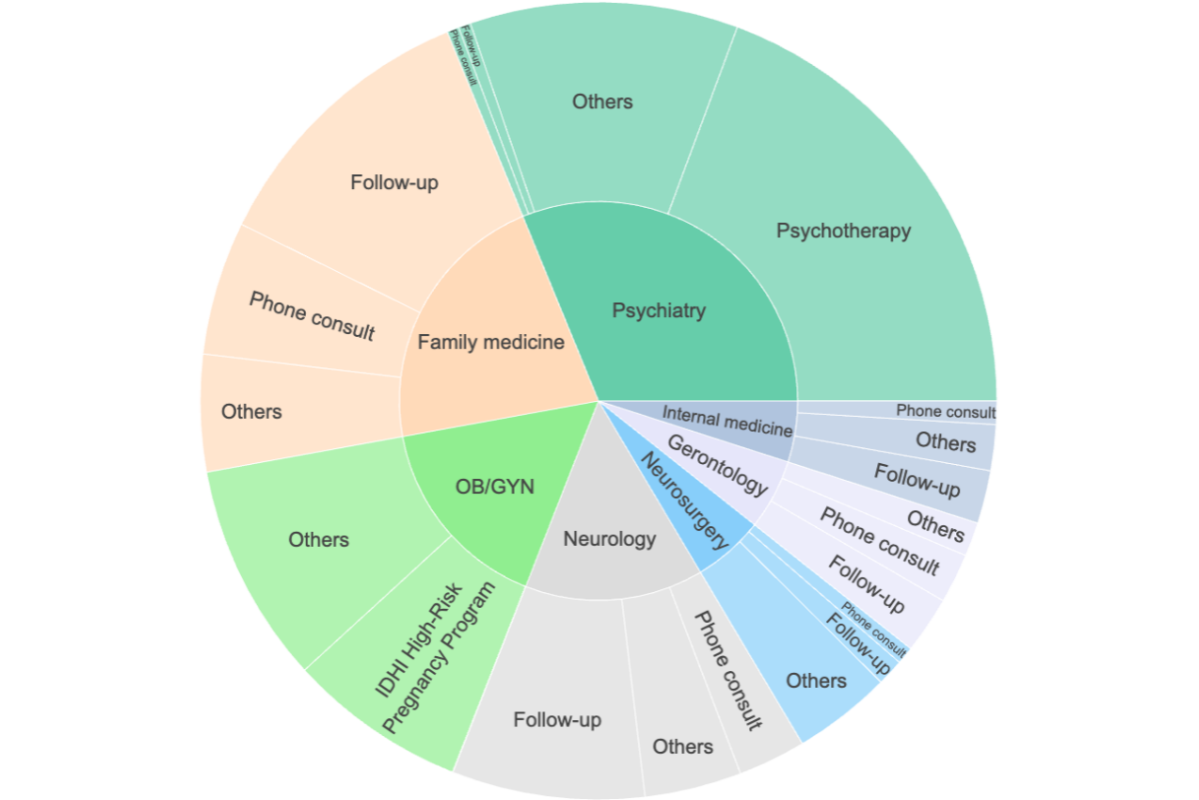
Appointment types of telehealth visits (2020-2022). IDHI: Institute for Digital Health & Innovation; OB/GYN: obstetrician and gynecologist.

In Figure 7, we provide a summary of the number of visits for different appointment types for various telehealth visits. Notably, except for *psychiatry* and *OB/GYN*, about 69.74% (39,270/56,307) of telehealth visits across all specialties were follow-up visits and phone conversations. The convenience of telehealth for follow-up visits is evident, as it saves patients the time and effort required to travel to health care facilities [27]. In addition, several studies have suggested that phone visits are more convenient for patients with inadequate broadband access or technological illiteracy [28,29].

Regarding *psychiatry* visits, approximately 61.95% (20,692/33,399) of visits focused on psychotherapy. This finding is consistent with other research that suggests telehealth visits as an alternative to in-person *psychiatry* visits, particularly for less common mental and physical health conditions [30,31].

#### In-Person Visits

**Figure 8:**
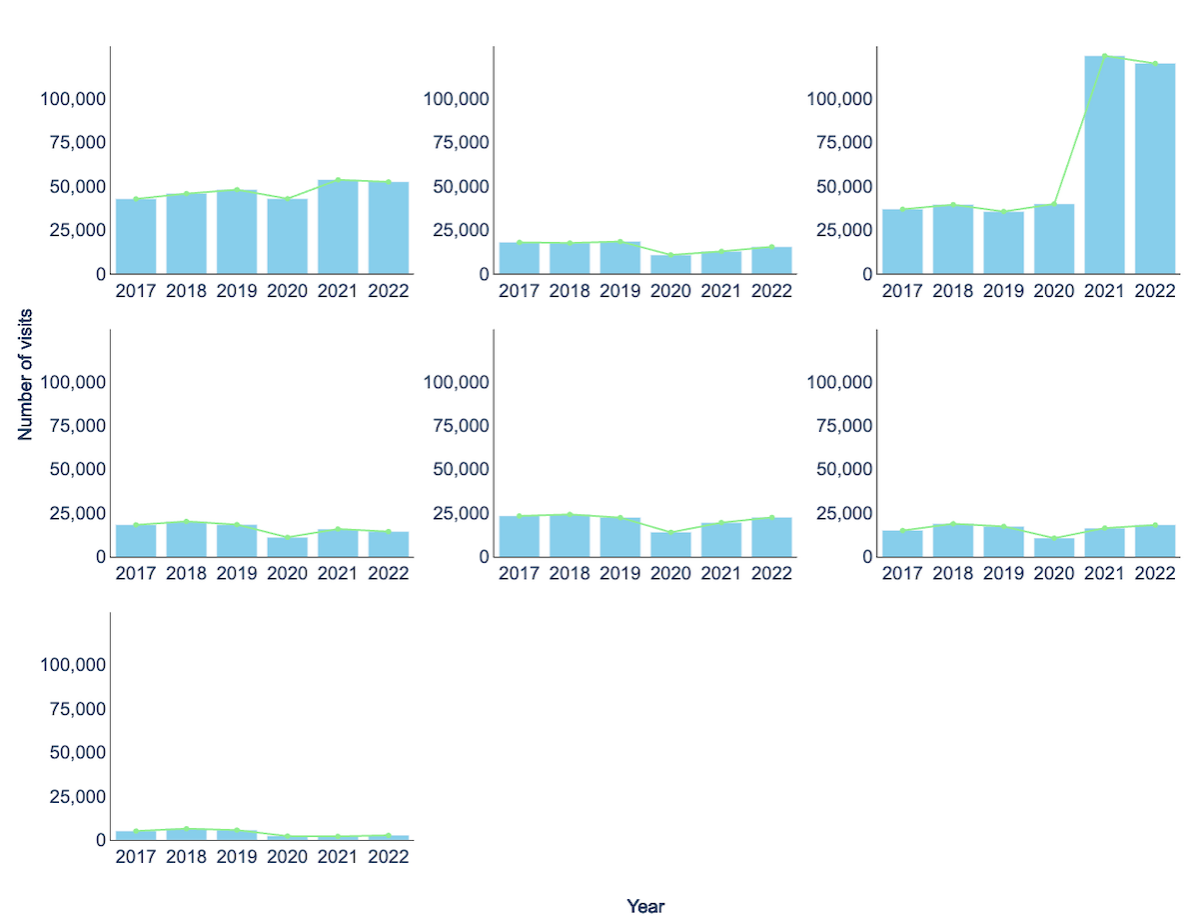
Number of visits for the selected health care specialties, call center and HealthNow (2017-2022) for in-person visits. OB/GYN: obstetrician and gynecologist.

Figure 8 provides the number of in-person visits at UAMS Health for 7 different specialties between 2017 and 2022. In 2020, the number of in-person visits decreased for all services except for *family medicine*. The low use of telehealth in *family medicine* may be due to physicians’ lack of training [32,33]. The number of in-person visits for *neurosurgery* went from 5605 in 2019 to 2184 in 2020. This corresponds to a 61% decrease. During the same period, the number of *psychiatry* visits went from 18,366 to 10,719, a 41.6% decrease, and the number of *gerontology* visits went from 18,393 to 11,140, a 39.4% decrease. The decrease in *neurosurgery* in-person visits resulted from the suspension of elective surgeries during the COVID-19 pandemic [34,35].

Because *psychiatry* visits do not require a physical examination, the decrease in the number of in-person visits observed is expected [34,36,37]. Telehealth visits are equivalent to in-person visits in terms of diagnostic accuracy, treatment effectiveness, quality of care, patient satisfaction, privacy, and confidentiality, as per the American Psychiatric Association (APA) [38]. Furthermore, the decrease in in-person *gerontology* services is likely due to the high vulnerability of older adults to COVID-19 infection [39].

In 2021, there was an increase in the number of in-person visits for all specialties except *neurosurgery*. However, recent studies suggest that most patients and health care providers preferred telehealth *neurosurgery* visits and were satisfied with the experience [34,40]. Overall, in 2022, we noticed minimal changes in the number of in-person visits, which indicates that demand for services is stabilizing. However, there were fewer total visits in 2022 compared to 2021, which included both in-person and telehealth visits. In summary, our observations suggest that patient visits shifted from in-person to telehealth during 2020 and 2021 compared with 2017 to 2019, with the exception of *OB/GYN*. When comparing 2022 to 2021, we observed only slight changes in the number of in-person and telehealth visits.

### Insurance Coverage

**Figure 3:**
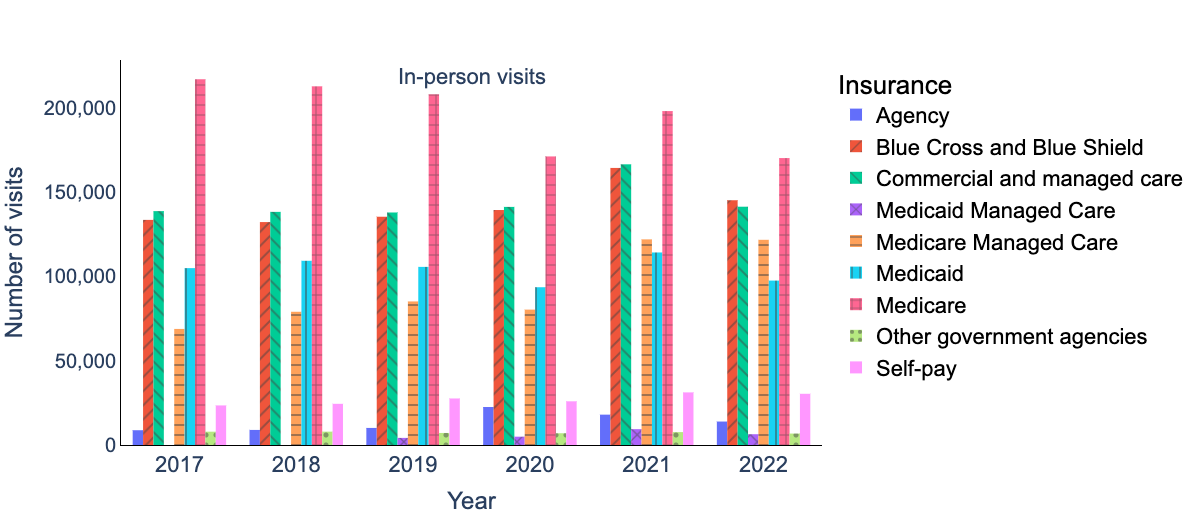
Insurance coverage breakdown (2017-2022) for in-person visits.

Most patients who used telehealth services during the period of 2020 to 2022 had their expenses covered by several insurance providers, including *Medicare*, *Blue Cross* and *Blue Shield*, *commercial and managed care*, *Medicaid*, and *Medicare Managed Care*. These insurance providers accounted for 92.9% (134,221/144,437) of all telehealth visits during this time, as shown in Figure 2. It is worth noting that most of the in-person visits were also covered by these same insurance providers, as depicted in Figure 3. Specifically, these insurance providers covered 91.8% (2,067,072/2,251,199) of all in-person visits that occurred between 2020 and 2022.

The mean values, SDs, and sample sizes (presented as n) of insurance-covered visits are indicated in Table 2. In addition, the result of the hypothesis tests and the corresponding *P* values are presented in Table 3. According to Table 3, the increase in insurance coverage for telehealth visits in 2020 compared to 2019 and in 2021 compared to 2020 was statistically significant. There were no statistically significant differences in insurance coverage for telehealth visits during 2021 and 2022. There were no statistically significant differences in insurance coverage for in-person visits during 2019 and 2020. However, the increase in insurance coverage for in-person visits in 2021 versus 2020 and the decrease in insurance coverage for in-person visits in 2022 versus 2021 were statistically significant.

**Table 2:** Descriptive statistics for insurance coverage (sample size: n=9 insurance providers).

Visit type	Visits (n) conducted in 2019 across insurance providers, mean (SD)			
	2019	2020	2021	2022
Telehealth	323 (376)	4372 (3781)	6145 (5227)	5530 (4544)
In-person	80,153 (72,629)	76,239 (64,081)	92,387 (76,280)	81,506 (66,888)

**Table 3:** Hypothesis testing results for insurance coverage for the average number of visits conducted across insurance providers in 2019, 2020, 2021, and 2022 (µ^2019^, µ^2020^, µ^2021^, and µ^2022^, respectively).

Visit type	*H*_*a*_ (*P* value)					
	*µ*^2019^ ≠ *µ*^2020^	*µ*^2019^ < *µ*^2020^	*µ*^2020^ ≠ *µ*^2021^	*µ*^2020^ < *µ*^2021^	*µ*^2021^ ≠ *µ*^2022^	*µ*^2021^ > *µ*^2022^
Telehealth	.004	.002	.004	.002	.06	—^e^
In-person	.43	—	.010	.007	.02	.001

^a^Not applicable.

During 2020 to 2022, telehealth visits covered by these insurance providers surged (Figure 2). *Medicare* telehealth visits increased by 19.3%, going from 9101 to 10,856. Telehealth visits covered by the *Blue Cross* and *Blue Shield* increased by 31.1%, going from 8042 to 10,542. Telehealth visits covered by *commercial and managed care* increased by 16%, growing from 8544 to 9908. Medicaid telehealth visits experienced a slight increase from 6889 to 7206, amounting to a 4.6% increase. *Medicare* *Managed Care* telehealth visits nearly doubled, increasing from 4013 to 7512, a notable 87.6% increase.

In contrast, in-person visits covered by these 5 insurance providers experienced different trends (Figure 3). During 2020 to 2022, in-person visits covered by *Medicare* decreased by 0.6%, going from 171,200 to 170,224. In-person visits covered by the *Blue Cross* and *Blue Shield* increased by 4.2%, going from 139,406 to 145,20. Similarly, in-person visits covered by *Medicaid* increased by 4.2%, going from 93,600 to 97,540. In-person visits covered by *commercial and managed care* increased by 0.1%, going from 141,257 to 141,368. *Medicare Managed Care* in-person visits saw a significant increase of 51.6%, going from 80,323 to 121,734.

**Figure 9:**
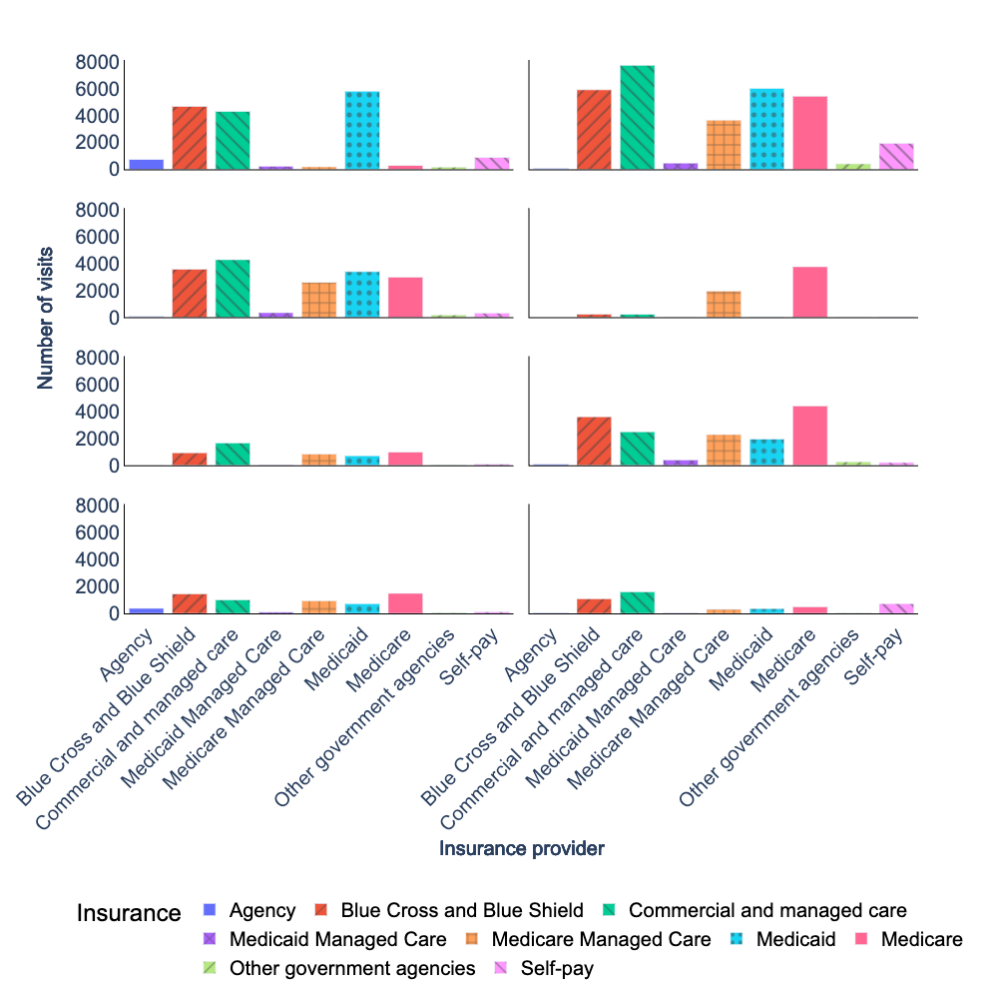
Insurance coverage of telehealth visits for the selected health care specialty, HealthNow (2020-2022). OB/GYN: obstetrician and gynecologist.

Figure 9 provides a summary of the number of telehealth visits categorized by the insurance provider and specialty from 2020 to 2022. Specifically in Arkansas, the *Medicare* plan covers adults aged >65 years and younger adults with a disability [41]. In *gerontology*, *neurology*, and *neurosurgery*, the highest number of visits was covered by *Medicare*, accounting for 60.81% (3754/6173), 28.14% (4409/15,667), and 24% (1484/6181), respectively. It is worth noting that a significant proportion of patients visiting these specialties are retirees and individuals with disabilities.

Moreover, in Arkansas, the *Medicaid* program covers pregnant women and children from low-income families [42]. Approximately 27% of Arkansas’ population is covered by *Medicaid* or *CHIP*, and approximately 35.67% (5312/14,892) of pregnant women who use *OB/GYN* services are unemployed [43]. This explains why *Medicaid* covers a large percentage (5797/17,120, 33.86%) of *OB/GYN* visits.

Our analysis revealed that approximately 15.58% (718/4607) of *HealthNow* visits, 6.07% (1913/31,516) of *psychiatry* visits, and 5% (858/17,130) of *OB/GYN* visits were self-pay. *HealthNow* services have proven attractive to patients who are willing to self-pay, as they offer accessible health care services 24/7 [22]. Patients also choose to self-pay for psychotherapy services due to limited coverage by insurance companies for certain psychiatric treatments [44]. In addition, UAMS Health offers uninsured patients a 60% discount, which may explain why patients opt to self-pay to receive essential prenatal care visits necessary for a healthy pregnancy [45,46].

### Appointment Scheduling

**Figure 10:**
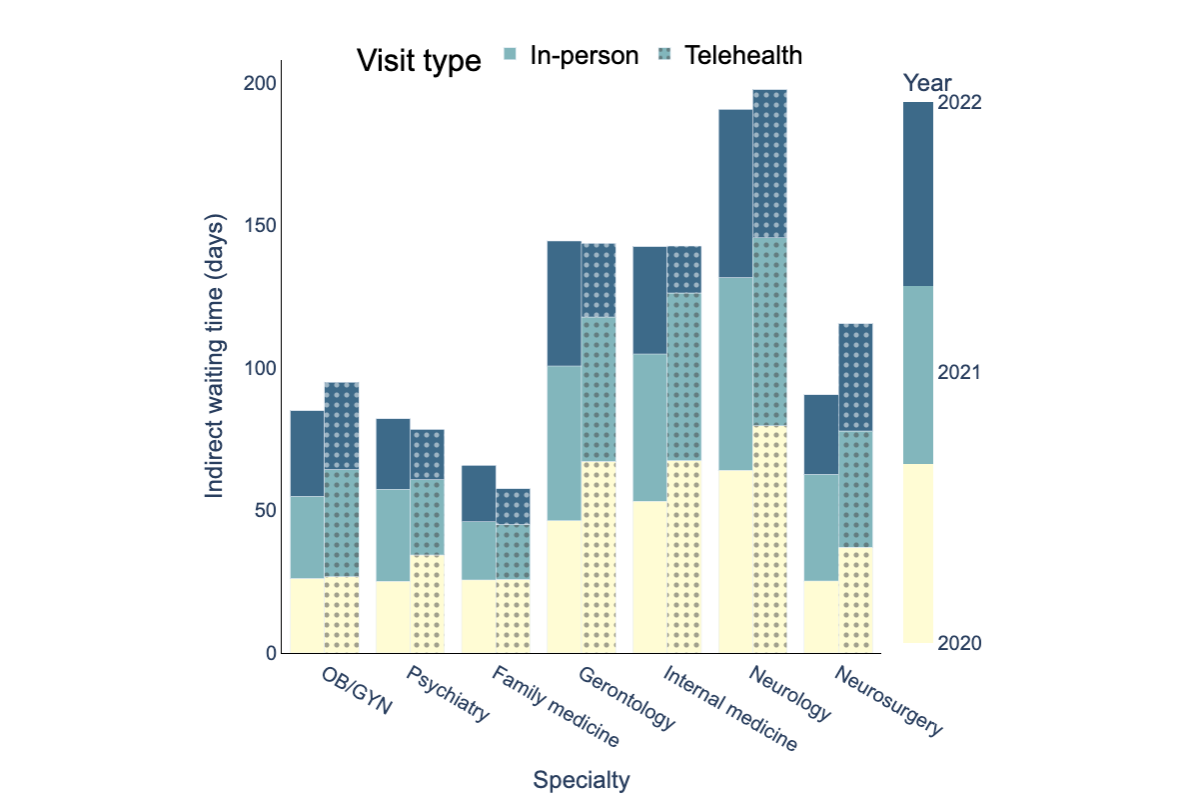
Indirect waiting time (days) for in-person and telehealth visits (2020-2022), OB/GYN: obstetrician and gynecologist.

**Figure 11:**
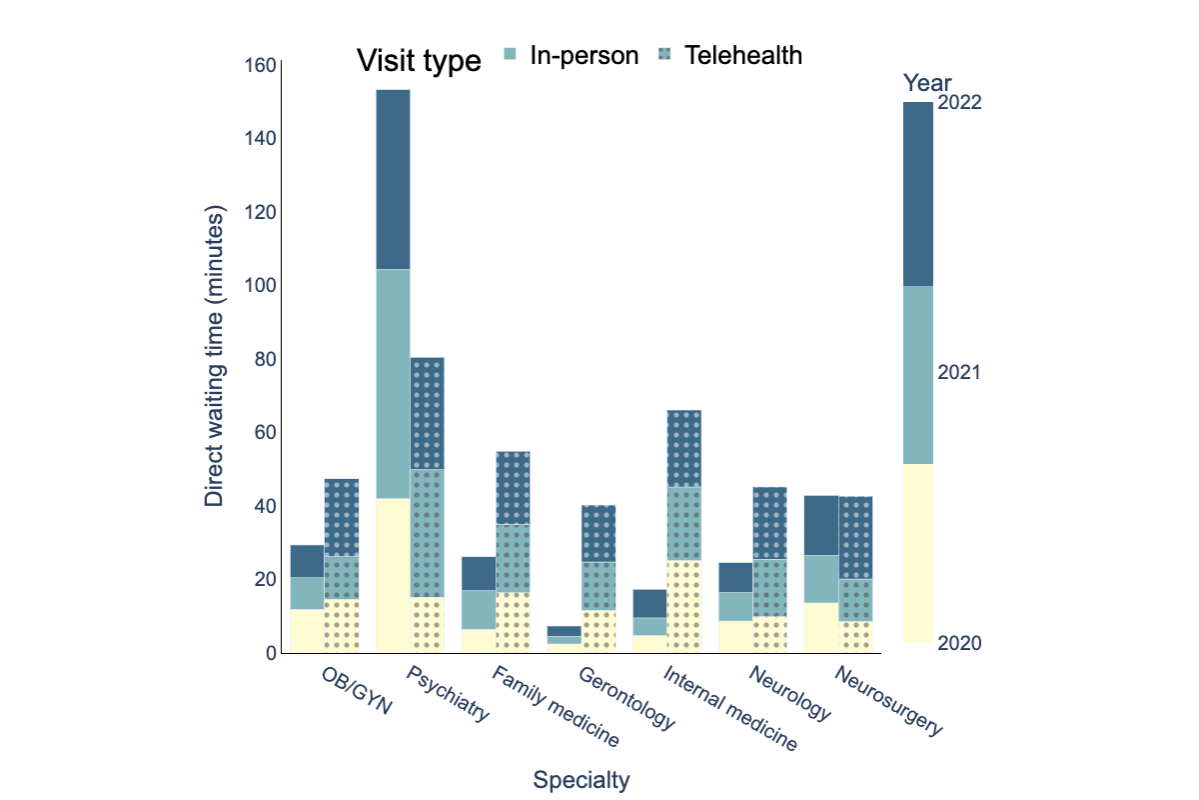
Direct waiting time (minutes) for in-person and telehealth visits (2020-2022), OB/GYN: obstetrician and gynecologist.

**Figure 12:**
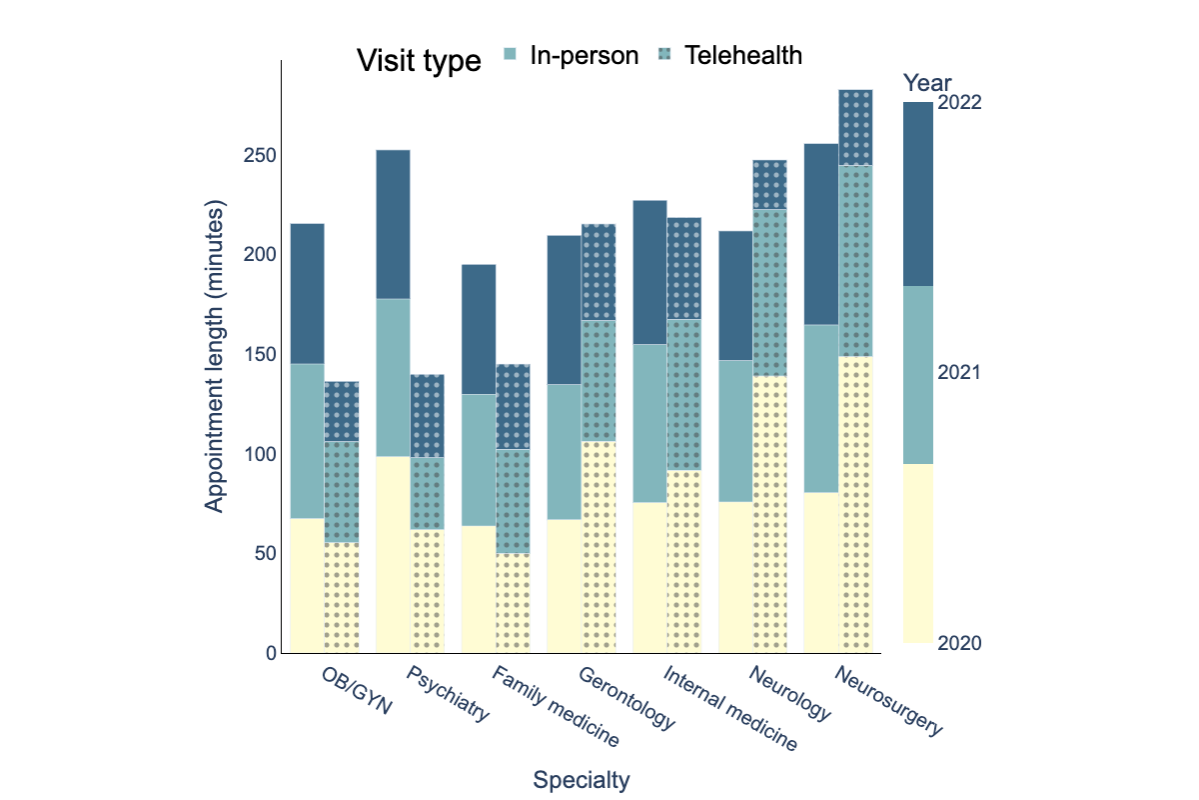
Appointment length (minutes) for in-person and telehealth visits (2020-2022), OB/GYN: obstetrician and gynecologist.

Figures 10-12 depict the average indirect waiting time, direct waiting time, and appointment length for each specialty for in-person and telehealth visits. In these figures, the bars with solid colors represent performance measures for in-person visits and the bars with patterns represent performance measures for telehealth visits. The mean values, SDs, and sample sizes (presented as *n*) of these appointment scheduling metrics are indicated in Tables 4-12. These metrics were obtained through an analysis of the processed data, as illustrated in Figure 5. The result of the hypothesis tests and the corresponding *P* values are also presented in Tables 4-12.

Table 4 compares indirect waiting times for in-person and telehealth visits by specialty for the years 2020, 2021, and 2022. Tables 7 and 10 present similar information but for direct waiting time and appointment length, respectively.

Table 4 examines whether there were statistically significant differences in indirect waiting times of in-person visits between 2021 and 2020 and between 2022 and 2021. Tables 8 and 11 are similar to Table 5 but for direct waiting times and appointment length, respectively. Tables 6, 9, and 12 evaluate whether there were statistically significant differences between 2021 and 2020 and between 2022 and 2021 in indirect waiting times, direct waiting times, and appointment length of telehealth visits, respectively.

UAMS Health experienced changes in average waiting times and appointment length for both telehealth and in-person visits between 2020 and 2022.

In 2020, the average indirect waiting time for telehealth visits for these specialties was 48.36 days, while the corresponding indirect waiting time for in-person visits was 37.99 days. In 2021, the average indirect waiting time for telehealth visits decreased by 5.6 days, and in 2022, it decreased even further by 15.2 days. However, in 2021, the average indirect waiting time for in-person visits increased by 3.78 days, and in 2022, it decreased by 7.05 days. Overall, the average indirect waiting time at UAMS Health in 2022 decreased (compared to 2020) by 20.8 days for telehealth visits and by 3.27 days for in-person visits.

Similarly, in 2020, the average direct waiting time for telehealth visits was 14.49 minutes, and the corresponding direct waiting time for in-person visits was 12.82 minutes. In 2021, the average direct waiting time for telehealth visits increased by 3.38 minutes, and in 2022, it increased further by 3.6 minutes. The average direct waiting time for in-person visits increased by 2.75 minutes in 2021 but decreased by 1 minute in 2022. As a result, the overall average direct waiting time at UAMS Health in 2022 increased by 6.99 minutes for telehealth visits and by 1.75 minutes for in-person visits.

Finally, in 2020, the average appointment length for telehealth visits was 93.24 minutes, and for in-person visits, it was 75.39 minutes. In 2021, the average appointment length for telehealth visits decreased by 28.3 minutes, and in 2022, it decreased by an additional 25.35 minutes. By contrast, the average appointment length for in-person visits decreased by 0.38 minutes in 2021 and further decreased by 1.64 minutes in 2022. Overall, the average appointment length at UAMS Health in 2022 decreased by 53.65 minutes for telehealth visits and by 2.02 minutes for in-person visits.

**Table 4:** Descriptive statistics and hypothesis testing results for mean indirect waiting time for in-person visits (μ^i,ind^) vs telehealth visits (μ^t,ind^)

Year and specialty			*μ* ^*i,ind*^						*μ* ^*t,ind*^						*H*_*a*_ (*P* value)		
			Minutes, mean (SD)			Visits, n		Minutes, mean (SD)				Visits, n		*μ*^*i,ind*^ ≠ *μ*^*t,ind*^			*μ*^*i,ind*^ > *μ*^*t,ind*^
**2020**																	
	Obstetrics and gynecology	26.1 (38.2)			33,580		26.8 (29.6)				6436		.21			—^a^	
	Psychiatry	25.2 (33.0)			7481		34.4 (36.8)				7459		*<*.001			.99	
	Family medicine	25.6 (42.5)			37,548		25.8 (41.2)				3539		.79			—	
	Gerontology	46.4 (64.8)			10,280		67.2 (67.9)				2144		*<*.001			.99	
	Internal medicine	53.3 (68.3)			13,198		67.5 (72.7)				1477		*<*.001			.99	
	Neurology	64.1 (60.0)			10,321		79.7 (65.0)				4100		*<*.001			.99	
	Neurosurgery	25.3 (46.7)			1750		37.1 (49.1)				2147		*<*.001			.99	
**2021**																	
	Obstetrics and gynecology	28.8 (39.7)			36,857		37.6 (36.8)				4918		*<*.001			.99	
	Psychiatry	32.3 (37.1)			9883		26.4 (32.2)				8595		*<*.001			*<*.001	
	Family medicine	20.4 (35.7)			120,737		19.2 (32.9)				11,386		.001			*<*.001	
	Gerontology	54.3 (70.0)			14,706		50.6 (67.0)				2163		.02			.01	
	Internal medicine	51.6 (70.9)			19,585		58.8 (73.0)				2195		*<*.001			.99	
	Neurology	67.6 (59.2)			16,361		66.0 (58.4)				6076		.07			—	
	Neurosurgery	37.4 (64.7)			2098		40.8 (41.9)				2525		.04			.98	
**2022**																	
	Obstetrics and gynecology	30.0 (31.5)		33,815				30.7 (27.5)		4043				.23			—
	Psychiatry	24.7 (30.4)		11,793				17.7 (23.4)		8303				*<*.001			*<*.001
	Family medicine	19.8 (33.1)		110,954				12.6 (23.8)		8143				*<*.001			*<*.001
	Gerontology	43.9 (56.5)		11,221				25.9 (42.4)		1709				*<*.001			*<*.001
	Internal medicine	37.7 (55.6)		18,705				16.5 (33.5)		1404				*<*.001			*<*.001
	Neurology	59.0 (53.4)		14,775				52.0 (53.6)		5054				*<*.001			*<*.001
	Neurosurgery	27.9 (36.4)		2383				37.7 (39.1)		1479				*<*.001			.99

^a^Not available.

**Table 5:** Descriptive statistics and hypothesis testing results for indirect waiting time for in-person visits for 2020 (μ^i,ind,20^), 2021 (μ^i,ind,21^), and 2022 (μ^i,ind,22^).

Specialty	*μ* ^*i,ind,20*^			*μ* ^*i,ind,21*^			*μ* ^*i,ind,22*^			*H*_*a*_ (*P* value)			
	Days, mean (SD)	Visits, n	Days, mean (SD)		Visits, n	Days, mean (SD)		Visits, n	*μ*^*i,ind,20*^ ≠ *μ*^*i,ind,21*^		*μ*^*i,ind,20*^ > *μ*^*i,ind,21*^	*μ*^*i,ind,21*^ ≠ *μ*^*i,ind,22*^	*μ^i,ind,21^ > μ^i,ind,22^*
Obstetrics and gynecology	26.1 (38.2)	33,580	28.8 (39.7)		36,857	30.0 (31.5)		33,815	*<*.001		.99	<.001	.99
Psychiatry	25.2 (33.0)	7481	32.3 (37.1)		9883	24.7 (30.4)		11,793	*<*.001		.99	<.001	<.001
Family medicine	25.6 (42.5)	37,548	20.4 (35.7)		120,737	19.8 (33.1)		110,954	*<*.001		*<*.001	<.001	<.001
Gerontology	46.4 (64.8)	10,280	54.3 (70.0)		14,706	43.9 (56.5)		11,221	*<*.001		.99	<.001	<.001
Internal medicine	53.3 (68.3)	13,198	51.6 (70.9)		19,585	37.7 (55.6)		18,705	*<*.001		.02	<.001	<.001
Neurology	64.1 (60.0)	10,321	67.6 (59.2)		16,361	59.0 (53.4)		14,775	*<*.001		.99	<.001	<.001
Neurosurgery	25.3 (46.7)	1750	37.4 (64.7)		2098	27.9 (36.4)		2383	.90		—^a^	<.001	<.001

^a^Not applicable.

**Table 6:** Descriptive statistics and hypothesis testing results for indirect waiting time for telehealth visits for 2020 (μ^t,ind,20^), 2021 (μ^t,ind,21^), and 2022 (μ^t,ind,22^).

Specialty	*μ* ^*t,ind,20*^		*μ* ^*t,ind,21*^			*μ* ^*t,ind,22*^			*H*_*a*_ (*P* value)			
	Days, mean (SD)	Visits, n	Days, mean (SD)	Visits, n	Days, mean (SD)		Visits, n	*μ* ^*t,ind,20*^ *≠ μ* ^*t,ind,21*^		*μ*^*t,ind,20*^ > *μ* ^*t,ind,21*^	*μ* ^*t,ind,21*^ *≠ μ* ^*t,ind,22*^	*μ* ^*t,ind,21*^ *> μ* ^*t,ind,22*^
Obstetrics and gynecology	26.8 (29.6)	6436	37.6 (36.8)	4918	30.7 (27.5)		4043	*<*.001		.99	*<*.001	*<*.001
Psychiatry	34.4 (36.8)	7459	26.4 (32.2)	8595	17.7 (23.4)		8303	*<*.001		*<*.001	*<*.001	*<*.001
Family medicine	25.8 (41.2)	3539	19.2 (32.9)	11,386	12.6 (23.8)		8143	*<*.001		*<*.001	*<*.001	*<*.001
Gerontology	67.2 (67.9)	2144	50.6 (67.0)	2163	25.9 (42.4)		1709	*<*.001		*<*.001	*<*.001	*<*.001
Internal medicine	67.5 (72.7)	1477	58.8 (73.0)	2195	16.5 (33.5)		1404	*<*.001		*<*.001	*<*.001	*<*.001
Neurology	79.7 (65.0)	4100	66.0 (58.4)	6076	52.0 (53.6)		5054	*<*.001		*<*.001	*<*.001	*<*.001
Neurosurgery	37.1 (49.1)	2147	40.8 (41.9)	2525	37.7 (39.1)		1479	.08		—^a^	.02	.01

^a^Not applicable.

**Table 7:** Descriptive statistics and hypothesis testing results for direct waiting time in-person (μ^i,dir^) and for telehealth (μ^t,dir^).

Year and specialty		*μ* ^*i,dir*^			*μ* ^*t,dir*^			*H*_*a*_ (*P* value)	
		Minutes, mean (SD)	Visits, n	Minutes, mean (SD)		Visits, n	*μ*^*i,dir*^ ≠ *μ*^*t,dir*^		*μ*^*i,dir*^ > *μ*^*t,dir*^
**2020**									
	Obstetrics and gynecology	11.9 (19.7)	40,859	14.6 (52.3)		4485	*<*.001		.99
	Psychiatry	42.0 (66.9)	2261	15.2 (47.8)		380	*<*.001		*<*.001
	Family medicine	6.4 (13.3)	33,312	16.4 (29.4)		1827	*<*.001		.99
	Gerontology	2.4 (10.4)	11,039	11.6 (38.7)		1530	*<*.001		.99
	Internal medicine	4.8 (19.2)	13,296	25.2 (47)		927	*<*.001		.99
	Neurology	8.7 (12.9)	10,027	9.9 (30)		2768	.002		.99
	Neurosurgery	13.6 (21.3)	2130	8.6 (36.1)		1683	*<*.001		*<*.001
**2021**									
	Obstetrics and gynecology	8.6 (21.5)	47,900	11.6 (32)		4004	*<*.001		.99
	Psychiatry	62.4 (81)	6219	34.7 (51.1)		5156	*<*.001		*<*.001
	Family medicine	10.5 (25.4)	101,967	18.5 (36.7)		8119	*<*.001		.99
	Gerontology	2.1 (8.6)	15,681	13.1 (27.8)		1782	*<*.001		.99
	Internal medicine	4.8 (16.5)	16,404	20.0 (38.2)		1738	*<*.001		.99
	Neurology	7.7 (11.3)	15,292	15.6 (30.3)		5250	*<*.001		.99
	Neurosurgery	12.9 (20.5)	1933	11.5 (33.1)		2328	.11		—^a^
**2022**									
	Obstetrics and gynecology	8.9 (20.9)	33,741	21.2 (23.6)		2335	*<*.001		.99
	Psychiatry	48.9 (68.33)	5744	30.6 (30.7)		10,990	*<*.001		*<*.001
	Family medicine	9.2 (17.5)	112,516	19.9 (36.4)		7552	*<*.001		.99
	Gerontology	2.8 (11)	13,932	15.5 (26.2)		1692	*<*.001		.99
	Internal medicine	7.8 (17.8)	20,248	21.0 (40)		1031	*<*.001		.99
	Neurology	8.2 (11.3)	16,725	19.7 (24.1)		5671	*<*.001		.99
	Neurosurgery	16.3 (27.3)	2507	22.6 (43.7)		1466	*<*.001		.99

^a^Not applicable.

**Table 8:** Descriptive statistics and hypothesis testing results for direct waiting time for in-person visits for 2020 (μ^i,dir,20^), 2021 (μ^i,dir,21^), and 2022 (μ^i,dir,22^).

Specialty	*μ* ^*i,dir,20*^			*μ* ^*i,dir,21*^			*μ* ^*i,dir,22*^			*H*_*a*_ (*P* value)			
	Minutes, mean (SD)	Visits, n	Minutes, mean (SD)		Visits, n	Minutes, mean (SD)		Visits, n	*μ*^*i,dir,20*^ ≠ *μ*^*i,dir,21*^		*μ*^*i,dir,20*^ > *μ*^*i,dir,21*^	*μ*^*i,dir,21*^ ≠ *μ*^*i,dir,22*^	*μ^i,ind,21^ > μ^i,ind,22^*
Obstetrics and gynecology	11.9 (19.7)	40,859	8.6 (21.5)		47,900	8.9 (20.9)		33,741	*<*.001		*<*.001	.02	.99
Psychiatry	42.0 (66.9)	2261	62.4 (81)		6219	48.9 (68.33)		5744	*<*.001		.99	*<*.001	*<*.001
Family medicine	6.4 (13.3)	33,312	10.5 (25.4)		101,967	9.2 (17.5)		112,516	*<*.001		.99	*<*.001	*<*.001
Gerontology	2.4 (10.4)	11,039	2.1 (8.6)		15,681	2.8 (11)		13,932	.006		*<*.001	*<*.001	.99
Internal medicine	4.8 (19.2)	13,296	4.8 (16.5)		16,404	7.8 (17.8)		20,248	.83		—^a^	*<*.001	.99
Neurology	8.7 (12.9)	10,027	7.7 (11.3)		15,292	8.2 (11.3)		16,725	*<*.001		*<*.001	.001	.99
Neurosurgery	13.6 (21.3)	2130	12.9 (20.5)		1933	16.3 (27.3)		2507	.28		—	*<*.001	.99

^a^Not applicable.

**Table 9:** Descriptive statistics and hypothesis testing results for direct waiting time for telehealth visits for 2020 (μ^t,dir,20^), 2021 (μ^t,dir,21^), and 2022 (μ^t,dir,22^).

Specialty	*μ* ^*t,dir,20*^		*μ* ^*t,dir,21*^			*μ* ^*t,dir,22*^			*H*_*a*_ (*P* value)			
	Minutes, mean (SD)	Visits, n	Minutes, mean (SD)	Visits, n	Minutes, mean (SD)		Visits, n	*μ*^*t,dir,20*^ ≠ *μ*^*t,dir,21*^		*μ*^*t,dir,20*^ > *μ*^*t,dir,21*^	*μ*^*t,dir,21*^ ≠ *μ*^*t,dir,22*^	*μ*^*t,dir,21*^ > *μ*^*t,dir,22*^
Obstetrics and gynecology	14.6 (52.3)	4485	11.6 (32.0)	4004	21.2 (23.6)		2335	.002		*<*.001	*<*.001	.99
Psychiatry	15.2 (47.8)	380	34.7 (51.1)	5156	30.6 (30.7)		10,990	*<*.001		.99	*<*.001	*<*.001
Family medicine	16.4 (29.4)	1827	18.5 (36.7)	8119	19.9 (36.4)		7552	.02		.99	.02	.99
Gerontology	11.6 (38.7)	1530	13.1 (27.8)	1782	15.5 (26.2)		1692	.19		—^a^	.01	.99
Internal medicine	25.2 (47.0)	927	20.0 (38.2)	1738	21.0 (40)		1031	.002		*<*.001	.53	—
Neurology	9.9 (30)	2768	15.6 (30.3)	5250	19.7 (24.1)		5671	*<*.001		.99	*<*.001	.99
Neurosurgery	8.6 (36.1)	1683	11.5 (33.1)	2328	22.6 (43.7)		1466	.007		.99	*<*.001	.99

^a^Not applicable.

**Table 10:** Descriptive statistics and hypothesis testing results for appointment length in-person (μ^i,app^) and for telehealth (μ^t,app^).

Year and specialty			*μ* ^*i,app*^				*μ* ^*t,app*^				*H*_*a*_ (*P* value)		
			Minutes, mean (SD)		Visits, n		Minutes, mean (SD)		Visits, n		*μ*^*i,app*^ ≠ *μ*^*t,app*^		*μ*^*i,app*^ > *μ*^*t,app*^
**2020**													
	Obstetrics and gynecology	67.4 (45.6)		40,246		55.3 (88.4)		99		.09		.004	
	Psychiatry	98.4 (87.7)		1769		61.8 (74.1)		63		.001		.001	
	Family medicine	63.7 (34.3)		33,423		50.0 (78.1)		960		*<*.001		*<*.001	
	Gerontology	66.8 (35.2)		9825		106.2 (100.4)		290		*<*.001		.99	
	Internal medicine	75.4 (43.3)		13,224		91.7 (123.1)		449		*<*.001		.99	
	Neurology	75.8 (48.8)		9911		139.0 (96.8)		2524		*<*.001		.99	
	Neurosurgery	80.3 (52.9)		2080		148.7 (90.1)		1523		*<*.001		.99	
**2021**													
	Obstetrics and gynecology	77.6 (51.6)		47,879		50.7 (91.2)		1051		*<*.001		*<*.001	
	Psychiatry	79.1 (85.6)		5105		36.2 (39.3)		2152		*<*.001		*<*.001	
	Family medicine	66.1 (39.2)		106,370		52.2 (76.2)		5535		*<*.001		*<*.001	
	Gerontology	67.8 (34)		13,741		60.6 (67.1)		1081		*<*.001		*<*.001	
	Internal medicine	79.3 (43.2)		16,466		75.7 (103)		1195		.02		.008	
	Neurology	70.9 (43.4)		15,247		83.4 (81.2)		4388		*<*.001		.99	
	Neurosurgery	84.2 (51.8)		1878		95.8 (74.3)		1682		*<*.001		.99	
**2022**													
	Obstetrics and gynecology	70.4 (38.1)		33,291		30.2 (71.7)		878		*<*.001		*<*.001	
	Psychiatry	74.8 (77.6)		5279		41.8 (38.1)		5907		*<*.001		*<*.001	
	Family medicine	65.2 (37.6)		110,065		42.7 (58.8)		6246		*<*.001		*<*.001	
	Gerontology	74.9 (38.6)		12,603		48.4 (51.4)		1517		*<*.001		*<*.001	
	Internal medicine	72.4 (38.6)		19,824		51.1 (66.6)		838		*<*.001		*<*.001	
	Neurology	65.0 (38.1)		16,635		24.9 (40.1)		3835		*<*.001		*<*.001	
	Neurosurgery	90.9 (53.7)		2436		38.0 (42.2)		782		*<*.001		*<*.001	

**Table 11:** Descriptive statistics and hypothesis testing results for mean appointment length for in-person visits for 2020 (μ^i,app,20^), 2021 (μ^i,app,21^), and 2022 (μ^i,app,22^).

Specialty	*μ* ^*i,app,20*^			*μ* ^*i,app,21*^			*μ* ^*i,app,22*^			*H*_*a*_ (*P* value)			
	Minutes, mean (SD)	Visits, n	Minutes, mean (SD)		Visits, n	Minutes, mean (SD)		Visits, n	*μ*^*i,app,20*^ ≠ *μ*^*i,app,21*^		*μ*^*i,app,20*^ > *μ*^*i,app,21*^	*μ*^*i,app,21*^ ≠ *μ*^*i,app,22*^	*μ*^*i,app,21*^ > *μ*^*i,app,22*^
Obstetrics and gynecology	67.4 (45.6)	40,246	77.6 (51.6)		47,879	70.4 (38.1)		33,291	*<*.001		1.0	*<*.001	*<*.001
Psychiatry	98.4 (87.7)	1769	79.1 (85.6)		5105	74.8 (77.6)		5279	*<*.001		*<*.001	.007	*<*.001
Family medicine	63.7 (34.3)	33,423	66.1 (39.2)		106,370	65.2 (37.6)		110,065	*<*.001		.99	*<*.001	*<*.001
Gerontology	66.8 (35.2)	9825	67.8 (34)		13,741	74.9 (38.6)		12,603	.02		.99	*<*.001	.99
Internal medicine	75.4 (43.3)	13,224	79.3 (43.2)		16,466	72.4 (38.6)		19,824	*<*.001		.99	*<*.001	*<*.001
Neurology	75.8 (48.8)	9911	70.9 (43.4)		15,247	65.0 (38.1)		16,635	*<*.001		*<*.001	*<*.001	*<*.001
Neurosurgery	80.3 (52.9)	2080	84.2 (51.8)		1878	90.9 (53.7)		2436	.02		.99	*<*.001	.99

**Table 12:** Descriptive statistics and hypothesis testing results for appointment length for telehealth visits for 2020 (μ^t,app,20^), 2021 (μ^t,app,21^), and 2022 (μ^t,app,22^).

Specialty	*μ* ^*t,app,20*^			*μ* ^*t,app,21*^			*μ* ^*t,app,22*^			*H*_*a*_ (*P* value)			
	Minutes, mean (SD)	Visits, n	Minutes, mean (SD)		Visits, n	Minutes, mean (SD)		Visits, n	*μ*^*t,app,20*^ ≠ *μ*^*t,app,21*^		*μ*^*t,app,20*^ > *μ*^*t,app,21*^	*μ*^*t,app,21*^ ≠ *μ*^*t,app,22*^	*μ*^*t,app,21*^ > *μ*^*t,app,22*^
Obstetrics and gynecology	55.3 (88.4)	99	50.7 (91.2)		1051	30.2 (71.7)		878	.63		—^a^	*<*.001	*<*.001
Psychiatry	61.8 (74.1)	63	36.2 (39.3)		2152	41.8 (38.1)		5907	*<*.001		*<*.001	*<*.001	.99
Family medicine	50.0 (78.1)	960	52.2 (76.2)		5535	42.7 (58.8)		6246	.41		—	*<*.001	*<*.001
Gerontology	106.2 (100.4)	290	60.6 (67.1)		1081	48.4 (51.4)		1517	*<*.001		*<*.001	*<*.001	*<*.001
Internal medicine	91.7 (123.1)	449	75.7 (103)		1195	51.1 (66.6)		838	.008		*<*.001	*<*.001	*<*.001
Neurology	139.0 (96.8)	2524	83.4 (81.2)		4388	24.9 (40.1)		3835	*<*.001		*<*.001	*<*.001	*<*.001
Neurosurgery	148.7 (90.1)	1523	95.8 (74.3)		1682	38.0 (42.2)		782	*<*.001		*<*.001	*<*.001	*<*.001

^a^Not applicable.

## Discussion

### Principal Findings

This study explores trends in telehealth use across specialties and insurance coverage in Arkansas by analyzing historical electronic medical records. We observed a significant increase in telehealth use in 2020 due to the COVID-19 pandemic. This increase is not uniform across different specialties. We focused our analysis on those specialties that have the highest number of telehealth visits. Most telehealth services during 2020 to 2022 were covered by *Medicare*, *Blue Cross* and *Blue Shield*, *commercial and managed care*, *Medicaid*, and *Medicare Managed Care*. We used 3 scheduling metrics (ie, indirect waiting time, direct waiting time, and appointment length) to highlight differences between in-person and telehealth services. Our analysis points to the potential benefits of telehealth in providing access to health care, particularly for patients who need psychiatric care.

The relaxation of regulations on remote communication technology for health care services by the Health and Human Services Office for Civil Rights due to the COVID-19 pandemic was one of the main factors that led to the increased use of telehealth during this period. This change in policy led to *Medicare* and *Medicaid* programs, as well as private insurance companies, expanding coverage for telehealth visits. Within the UAMS Health system, during 2020 to 2022, 92.9% (134,221/144,437) of telehealth patients were covered by *Medicare*, *Blue Cross* and *Blue Shield*, *commercial and managed care*, *Medicare*, and *Medicare Managed Care*, which also covered 91.8% (2,067,072/2,251,199) of in-person visits during this period. As a result, telehealth was more commonly used for specialties, such as *OB/GYN*, *psychiatry*, *family medicine*, *gerontology*, *internal medicine*, *neurology*, and *neurosurgery*. Before 2020, *OB/GYN* patients used telehealth visits covered by the ANGELS program, which started in 2003 and provided telemedicine consultations to pregnant patients considered high risk at UAMS.

Since 2020, UAMS Health has been providing both in-person and telehealth visits. Initially, the transition to telehealth caught health care facilities unprepared and employees were not trained, resulting in longer average indirect waiting times and appointment lengths for telehealth visits. In 2020, telehealth visits had an average indirect waiting time that was 10.37 days longer than in-person visits, an average direct waiting time that was 1.66 minutes longer, and an appointment length that was 17.85 minutes longer.

However, in 2021 and 2022, UAMS Health made significant improvements to its telehealth service. The average indirect waiting time decreased by 20.8 days for telehealth visits and 3.27 days for in-person visits compared to 2020, and the average appointment length decreased by 53.65 minutes for telehealth visits and 2.02 minutes for in-person visits. These improvements could be attributed to the flexibility that telehealth visits provide in using valuable resources, such as staff and physician time. These improvements could as well be attributed to the heightened adaptability of patients to technology and telehealth. In addition, *HealthNow* was newly established in January 2020 and may have improved in many ways as program staff and health care providers gained additional experience.

Although the decrease in appointment length might be due to improved usability and familiarity with the service, shorter appointment time may be a cause for concern because patients have less time with their doctors. This could negatively impact the quality of care provided. In addition, the average direct waiting time increased by 6.99 minutes for telehealth visits and 1.75 minutes for in-person visits in 2022.

Overall, UAMS Health has made significant strides in improving its telehealth service since its initial implementation in 2020. The decrease in waiting times and appointment length is a positive development, although the increase in direct waiting times and the potential impact on the quality of care provided require further monitoring.

From 2020 to 2022, *psychiatry* had the highest number of telehealth visits among the different specialties that offer this service. According to the APA, telehealth *psychiatry* visits are just as accurate as in-person visits in terms of diagnosis, treatment effectiveness, quality of care, patient satisfaction, privacy, and confidentiality [38]. This is why the APA and the American Telemedicine Association collaborated to develop best practices guidelines for telemental health delivery [47].

Our study demonstrates that the appointment scheduling metrics for *psychiatry* telehealth visits are shorter than those for in-person visits. This suggests that patients would benefit from continued access to and reimbursement for telehealth *psychiatry* visits. Overall, the high number of telehealth *psychiatry* visits and their comparable diagnostic accuracy and treatment effectiveness to in-person visits highlight the value of telehealth in this field. The APA’s best practices guidelines and the shorter appointment scheduling metrics for telehealth *psychiatry* visits provide further support for the continued use and reimbursement of this service.

In 2020, there was a significant decrease in the number of in-person visits for patients covered by *Medicare*. However, in 2021, we observed a trend of increased in-person visits for all patients, including those covered by *Medicare*. Despite this, the number of in-person visits covered by *Medicare* remained lower than that in 2019, which was not the case for visits covered by other insurance providers. In 2022, there was a decrease in the number of in-person visits covered by *Medicare*, *Medicaid*, and *other government agencies* compared to 2019.

These findings indicate that telehealth visits have improved health care accessibility in Arkansas. Many patients continued to use telehealth visits in 2022, even after the end of the COVID-19 public health emergency. Therefore, we suggest that insurance companies and policy makers take note of these observations and continue to cover these services. Telehealth services are vital for patients in Arkansas, especially because 59 out of the total 75 counties are classified as medically underserved and another 15 are classified as partially underserved [48]. These classifications are due to a shortage of primary care health service providers in Arkansas, making it challenging for patients to visit health care providers. This shortage is particularly pressing for patients living in rural areas, people on *Medicaid*, and children in ARKids [49].

Providing access to health care through telehealth reduces these vulnerabilities, making it a crucial aspect of health care in Arkansas. Thus, moving forward, insurance companies will need to (1) rethink their reimbursement policies to ensure payment equity between telehealth and in-person visits [50,51] and (2) change policies, such as lift restrictions on new patients.

Our study used a large amount of data to evaluate essential appointment scheduling metrics for both telehealth and in-person visits, including direct and indirect waiting times and appointment length. In 2022, we found that the average indirect waiting time for telehealth visits was 7.17 days shorter than for in-person visits, while the average direct waiting time was 6.89 minutes longer. In addition, the appointment length was 33.78 minutes shorter for telehealth visits than that for in-person visits. This could be attributed to telehealth visits not requiring a physical room, making them easier to schedule and resulting in shorter indirect waiting times.

Although longer direct waiting times may not be as concerning for telehealth visits, the shorter appointment length may be an area of concern. Further research is needed to identify the reasons behind this difference.

While many recent studies have analyzed telehealth use [2,3,52-57], only a few have focused on evaluating appointment scheduling metrics [58-62]. Previous studies have primarily focused on appointment adherence rather than appointment duration and waiting time. Our study provides crucial insights into comparing the efficiency of telehealth and in-person visits and can motivate changes in current practices to improve patient experience.

### Limitations

Although our study provides valuable insights into telehealth visits for UAMS Health, it has a few limitations that should be acknowledged. First, the results of our analysis may not be generalizable to other health care providers in Arkansas, as their patient populations and care settings may differ. Nonetheless, our research framework can be applied to other health care providers to assess their telehealth visits. Second, our study used data from multiple health care providers at UAMS Health, with some offering only telehealth services and others providing a mix of in-person and telehealth visits. Therefore, our results reflect an overall evaluation of telehealth and in-person visits for all health care providers at UAMS Health and may not be representative of individual health care providers. Finally, we excluded records with incomplete or inaccurate time stamps from our analysis, which may have limited our sample size and introduced bias in our metrics calculations. Future research could address these limitations by using more comprehensive and accurate datasets and by comparing the performance of telehealth visits across different health care providers and patient populations.

### Conclusions

In 2020, telehealth services in the United States saw an uptick due to policy changes allowing insurance companies to reimburse for these services. Most telehealth services during 2020 to 2022 were covered by *Medicare*, *Blue Cross* and *Blue Shield*, *commercial and managed care*, *Medicaid*, and *Medicare Managed Care*. Our analysis of electronic medical records from 2017 to 2022 collected by UAMS Health shows that telehealth use increased in Arkansas. This increase in telehealth services provided better health care access to individuals living in medically underserved and rural areas.

Our observations indicate that there was a shift in patient visits from in-person to telehealth visits during 2020 and 2021 for *psychiatry*, *family medicine*, *gerontology*, *internal medicine*, *neurology*, and *neurosurgery* visits. However, we did not observe this shift for *OB/GYN patients*. The reason for this is because pregnant women had access to telehealth before 2020 through the ANGELS program, which was established in 2003 and offered telemedicine consultations to UAMS patients in high-risk pregnancies.

When comparing the data from 2022 to 2021, we noticed only minor differences in the number of telehealth and in-person visits. Our findings indicate that *psychiatry* had the highest number of telehealth visits, followed by *OB/GYN* and *family medicine*. Notably, *psychiatry* visits do not require a physical examination and offer comparable diagnostic accuracy, treatment effectiveness, quality of care, patient satisfaction, privacy, and confidentiality as in-person visits.

To evaluate the efficiency of current telehealth appointment scheduling processes at UAMS Health, we compared the performance of telehealth and in-person visits using direct and indirect waiting times and appointment length. Our analysis of these metrics can inform improvements to current practices and motivate changes to optimize the scheduling of telehealth visits.

### Future Directions

The scope of our research is impacted by the data available. We were not able to delve deeper in our analysis of telehealth use within each specialty because we did not have access to cultural, demographic, and socioeconomic data about patients. Researchers who have access to such data could use the proposed framework to determine what factors influence patients’ decisions to use telehealth.

Our research is limited to the state of Arkansas. The scope of this research can be extended by investigating telehealth use in other states. Such an analysis would determine how the distribution of population between urban and rural areas, access to broadband networks, access to health care, and health care resources available do impact the use of telehealth.

Our study uses 3 metrics to evaluate the performance of telehealth and in-person visits, which are direct and indirect waiting time and appointment duration. Researchers who have access to data about resources available in a health care facility (the number of physicians or nurses and their schedules, the number of rooms, etc) could calculate other relevant metrics, such as resource use rate for in-person and telehealth visits. This information is important to design strategies (develop schedules) for the optimal use of resources and to provide high-quality service to in-person and telehealth patients.

## Abbreviations

ANGELS: Antenatal and Neonatal Guidelines, Education and Learning System
APA: American Psychiatric Association
OB/GYN: obstetrics and gynecology
UAMS: University of Arkansas for Medical Sciences
UAMS Health: University of Arkansas for Medical Sciences Medical Center

## Data Availability

The datasets generated and analyzed during this study are not publicly available due to the use of medical data from a health care organization.

## References

[1] (2022). Declarations of a public health emergency. US Department of Health & Human Services.

[2] Koonin LM, Hoots B, Tsang CA, Leroy Z, Farris K, Jolly B, Antall P, McCabe B, Zelis CB, Tong I, Harris AM (2020). Trends in the use of telehealth during the emergence of the COVID-19 pandemic - United States, January-March 2020. MMWR Morb Mortal Wkly Rep.

[3] Demeke HB, Pao LZ, Clark H, Romero L, Neri A, Shah R, McDow KB, Tindall E, Iqbal NJ, Hatfield-Timajchy K, Bolton J, Le X, Hair B, Campbell S, Bui C, Sandhu P, Nwaise I, Armstrong PA, Rose MA (2020). Telehealth practice among health centers during the COVID-19 pandemic - United States, July 11-17, 2020. MMWR Morb Mortal Wkly Rep.

[4] Baum A, Kaboli PJ, Schwartz MD (2021). Reduced in-person and increased telehealth outpatient visits during the COVID-19 pandemic. Ann Intern Med.

[5] Wosik J, Fudim M, Cameron B, Gellad ZF, Cho A, Phinney D, Curtis S, Roman M, Poon EG, Ferranti J, Katz JN, Tcheng J (2020). Telehealth transformation: COVID-19 and the rise of virtual care. J Am Med Inform Assoc.

[6] Maugeri A, Barchitta M, Basile G, Agodi A (2024). Public and research interest in telemedicine from 2017 to 2022: infodemiology study of Google trends data and bibliometric analysis of scientific literature. J Med Internet Res.

[7] (2020). Medicare telemedicine health care provider fact sheet. Centers for Medicare & Medicaid Services and others.

[8] Telehealth: delivering care safely during COVID-19. US Department of Health and Human Services.

[9] Adler-Milstein J, Kvedar J, Bates DW (2014). Telehealth among US hospitals: several factors, including state reimbursement and licensure policies, influence adoption. Health Aff (Millwood).

[10] (2020). Telehealth policy changes after the COVID-19 public health emergency. US Department of Health and Human Services.

[11] COVID-19 public health emergency (PHE). US Department of Health and Human Services.

[12] Cubanski J, Kates J, Tolbert J What happens when COVID-19 emergency declarations end? Implications for coverage, costs, and access. KFF.

[13] (2021). What happens to telemedicine after COVID-19?. Association of American Medical Colleges.

[14] Mallow J, Davis S Health insurers are starting to roll back coverage for telehealth – even though demand is way up due to COVID-19. PBS News.

[15] Adams K 10 biggest barriers to telehealth, as told by physicians. Becker's Healthcare.

[16] Andrews E, Berghofer K, Long J, Prescott A, Caboral-Stevens M (2020). Satisfaction with the use of telehealth during COVID-19: an integrative review. Int J Nurs Stud Adv.

[17] Odebunmi OO, Hughes TD, Waters AR, Urick BY, Herron C, Wangen M, Rohweder C, Ferrari RM, Marciniak MW, Wheeler SB, Brenner AT, Shah PD (2024). Findings from a national survey of older US adults on patient willingness to use telehealth services: cross-sectional survey. J Med Internet Res.

[18] Schriger SH, Klein MR, Last BS, Fernandez-Marcote S, Dallard N, Jones B, Beidas RS (2022). Community mental health clinicians' perspectives on telehealth during the COVID-19 pandemic: mixed methods study. JMIR Pediatr Parent.

[19] Phenicie R, Acosta Wright R, Holzberg J (2021). Patient satisfaction with telehealth during COVID-19: experience in a rural county on the United States-Mexico border. Telemed J E Health.

[20] (2022). UAMS physician's call center. UAMS Institute for Digital Health & Innovation.

[21] Boulden B Now offers 24/7 digital health, live video access to convenient care. UAMS News.

[22] Digital health and innovation. UAMSHealth.

[23] Bain BZ, Engelhardt M (1992). Introduction to Probability and Mathematical Statistics. Volume 4.

[24] Hailemeskel Abebe TH (2019). The derivation and choice of appropriate test statistic (Z, t, F and Chi-Square test) in research methodology. Math Lett.

[25] Lowery C, Bronstein J, McGhee J, Ott R, Reece EA, Mays GP (2007). ANGELS and University of Arkansas for Medical Sciences paradigm for distant obstetrical care delivery. Am J Obstet Gynecol.

[26] Melchionna M New data shows telehealth usage drops by 4% nationally. TechTarget.

[27] Donelan K, Barreto EA, Sossong S, Michael C, Estrada JJ, Cohen AB, Wozniak J, Schwamm LH (2019). Patient and clinician experiences with telehealth for patient follow-up care. Am J Manag Care.

[28] Verma S (2020). Early impact of CMS expansion of Medicare telehealth during COVID-19. Health Affairs Forefront.

[29] Jaklevic MC (2020). Telephone visits surge during the pandemic, but will they last?. JAMA.

[30] Greenwood H, Krzyzaniak N, Peiris R, Clark J, Scott AM, Cardona M, Griffith R, Glasziou P (2022). Telehealth versus face-to-face psychotherapy for less common mental health conditions: systematic review and meta-analysis of randomized controlled trials. JMIR Ment Health.

[31] Krzyzaniak N, Greenwood H, Scott AM, Peiris R, Cardona M, Clark J, Glasziou P (2021). The effectiveness of telehealth versus face-to face interventions for anxiety disorders: a systematic review and meta-analysis. J Telemed Telecare.

[32] Moore MA, Coffman M, Jetty A, Petterson S, Bazemore A (2016). Only 15% of FPs report using telehealth; training and lack of reimbursement are top barriers. Am Fam Physician.

[33] Rangachari P, Mushiana SS, Herbert K (2021). A narrative review of factors historically influencing telehealth use across six medical specialties in the United States. Int J Environ Res Public Health.

[34] De Biase G, Freeman WD, Bydon M, Smith N, Jerreld D, Pascual J, Casler J, Hasse C, Quiñones-Hinojosa A, Abode-Iyamah K (2020). Telemedicine utilization in neurosurgery during the COVID-19 pandemic: a glimpse into the future?. Mayo Clin Proc Innov Qual Outcomes.

[35] (2020). COVID-19: guidance for triage of non-emergent surgical procedures. American College of Surgeons.

[36] Kane CK, Gillis K (2018). The use of telemedicine by physicians: still the exception rather than the rule. Health Aff (Millwood).

[37] Patel SY, Mehrotra A, Huskamp HA, Uscher-Pines L, Ganguli I, Barnett ML (2021). Variation in telemedicine use and outpatient care during the COVID-19 pandemic in the United States. Health Aff (Millwood).

[38] (2020). What is telepsychiatry?. American Psychiatric Association.

[39] Doraiswamy S, Jithesh A, Mamtani R, Abraham A, Cheema S (2021). Telehealth use in geriatrics care during the COVID-19 pandemic-a scoping review and evidence synthesis. Int J Environ Res Public Health.

[40] Nie JZ, Karras CL, Texakalidis P, Trybula SJ, Dahdaleh NS (2022). A systematic review of outpatient telemedicine use in neurosurgery since the start of coronavirus disease 2019. World Neurosurg.

[41] Norris L (2022). Medicare in Arkansas. Health Insurence.

[42] (2022). Health care programs. Arkansas Department of Human Services.

[43] Medicaid state fact sheets. Kaiser Family Foundation.

[44] Benjenk I, Chen J (2020). Trends in self-payment for outpatient psychiatrist visits. JAMA Psychiatry.

[45] (2023). UAMS financial assistance. UAMSHealth.

[46] (2017). What is prenatal care and why is it important?. National Institutes of Child Health and Human Development.

[47] Shore JH, Yellowlees P, Caudill R, Johnston B, Turvey C, Mishkind M, Krupinski E, Myers K, Shore P, Kaftarian E, Hilty D (2018). Best practices in videoconferencing-based telemental health April 2018. Telemed J E Health.

[48] (2023). What is shortage designation?. HRSA Health Workforce.

[49] Norris L (2021). Addressing Arkansas's health services shortages by empowering nurse practitioners. Arkansas Center for Research in Economics UoCA.

[50] Shachar C, Engel J, Elwyn G (2020). Implications for telehealth in a postpandemic future: regulatory and privacy issues. JAMA.

[51] (2022). Fiscal considerations for the future of telehealth. Committee for a Responsible Federal Budget.

[52] Howie F, Kreofsky BL, Ravi A, Lokken T, Hoff MD, Fang JL (2022). Rapid rise of pediatric telehealth during COVID-19 in a large multispecialty health system. Telemed J E Health.

[53] Mishra S, Dhuna N, Lancki N, Yeh C, Larson DN (2022). Telehealth utilization and patient satisfaction in an ambulatory movement disorders center during the COVID-19 pandemic. J Telemed Telecare.

[54] Haider Z, Aweid B, Subramanian P, Iranpour F (2020). Telemedicine in orthopaedics during COVID-19 and beyond: a systematic review. J Telemed Telecare.

[55] Schulz T, Long K, Kanhutu K, Bayrak I, Johnson D, Fazio T (2020). Telehealth during the coronavirus disease 2019 pandemic: rapid expansion of telehealth outpatient use during a pandemic is possible if the programme is previously established. J Telemed Telecare.

[56] Siddiqui S, Farr E, Dusto N, Chen L, Kocherginsky M, Skelton F, Verduzco-Gutierrez M, Lee S (2023). Telemedicine use among physiatrists during the early phase of the COVID-19 pandemic and potential for future use. Telemed J E Health.

[57] Fu R, Sutradhar R, Li Q, Eskander A (2022). Virtual and in-person visits by Ontario physicians in the COVID-19 era. J Telemed Telecare.

[58] Jeganathan S, Prasannan L, Blitz MJ, Vohra N, Rochelson B, Meirowitz N (2020). Adherence and acceptability of telehealth appointments for high-risk obstetrical patients during the coronavirus disease 2019 pandemic. Am J Obstet Gynecol MFM.

[59] Childs AW, Bacon SM, Klingensmith K, Li L, Unger A, Wing AM, Fortunati F (2021). Showing up is half the battle: the impact of telehealth on psychiatric appointment attendance for hospital-based intensive outpatient services during COVID-19. Telemed J E Health.

[60] Zoran S, Turcott C, Whitehead A, Hrabik L, Harris A, Scott Schwoerer J (2021). Rapid transition to telemedicine during the COVID-19 pandemic: medical genetics experience. WMJ.

[61] Snoswell CL, Comans TA (2021). Does the choice between a telehealth and an in-person appointment change patient attendance?. Telemed J E Health.

[62] Adams AM, Wu H, Zhang FR, Wajsberg JR, Bruney TL (2023). Postpartum care in the time of COVID-19: the use of telemedicine for postpartum care. Telemed J E Health.

